# Domestic

**Published:** 1840-11-14

**Authors:** 


### DOMESTIC.

*Stramonium in Dysmenorrhœa.* By Dr. T. J. COGLEY, of Mount Vernon, Ohio.—Mrs. S., æt. seventeen, commenced menstruating at fourteen. Had not the slightest difficulty during the first three periods; but during the fourth period she was exposed to cold, and the discharge was checked. From this time she was much afflicted with dysmenorrhœa at each period; but married soon after she was fifteen. This event seemed to augment the disease very much, and violent hysteria soon supervened, before each menstrual period. Every few months she had an attack of hysterical *convulsions*, and when she had no convulsions, experienced extreme difficulty in menstruating. She did not come under my notice until the 12th of May, 1840, when I found her labouring under all the distressing symptoms of dysmenorrhœa. I prescribed camphor in ten grain doses every hour, for three hours, without the least mitigation of the pain, which was excruciatingly severe. I next resorted to sulphate of morphia, the warm bath, and venesection; but, although the morphia was given in large and repeated doses, she continued to have the most violent convulsions I have ever witnessed for forty-eight hours, when I commenced the administration of stramonium. I gave the pulverized seeds, in large doses every four hours, until her pupils were greatly dilated, when the convulsions began to subside, and the menstrual discharge became more copious. During this administration I prescribed frequent enemata of simple warm water. Her senses speedily returned, and in a few days she was in ordinary health.

Believing that irritation existed in the cervical vertebræ, some time after the above attack I cupped her, on the back of the neck and between the shoulders, and afterwards applied an epispastic. About five days before the return of the menses in June, I gave her an active cathartic of ex. col. comp., after the operation of which I again put her upon the use of the stramonium in substance, in such doses as to dilate the pupils considerably. The patient was kept in this condition, on low diet, until the catamenia returned, which was about five weeks from the last period. The discharge was more copious, and attended with less pain, than at any former period, except the three first above mentioned. During the next interval the patient was kept on low diet, and a short time before the expected return of the menses, in July, she was bled for pain in the head, and again put under the influence of the stramonium as above. The discharge came on this time in about thirty-one days, and was attended with but little discomfort. It may be thought, that the venesection, cupping, blistering and cathartics, effected as much, perhaps, as the stramonium. But I think not; as they had been frequently prescribed, on former occasions, by other physicians. Before she came under my notice, the tinct. guiacum, oil of savin, and various other articles had been ineffectually tried, but particularly the first.

Having used the stramonium with more or less success, in at least six cases, I am induced to look upon it as one of the most efficient remedies we have in dysmenorrhœa. But the system must be prepared for its reception. It being, in my opinion, contra-indicated wherever there is a plethoric, or an inflammatory condition. These preparatory measures may be thought to have had some agency in ameliorating the disease, but I have often seen them fail, while on the other hand I have used the stramonium without its being preceded by venesection or catharsis. Where there is a plethoric state of the system it undoubtedly enhances its sanative effects, to premise venesection and catharsis, particularly the latter. It must be prescribed as above, at each successive menstrual period, until the dysmenorrhœa is entirely removed, or the woman becomes pregnant.—*Western Journal of Med. and Surg.*

